# Navigating the Uncharted Territory of Pediatric-Onset Multiple Sclerosis in a 12-Year-Old Male: A Case Study

**DOI:** 10.7759/cureus.56172

**Published:** 2024-03-14

**Authors:** Han Grezenko, Imam A Shah, Astri Anindya Sariza, Amna B Baluch, Ateesh Kumar, Muhammad Abubakar

**Affiliations:** 1 Translational Neuroscience, Barrow Neurological Institute, Phoenix, USA; 2 Internal Medicine, Chandka Medical College, Larkana, PAK; 3 Internal Medicine, Sriwijaya University, Palembang, IDN; 4 Internal Medicine, Universidad Autónoma de Guadalajara, Guadalajara, MEX; 5 Internal Medicine, Civil Hospital Karachi, Karachi, PAK; 6 Internal Medicine, Wah Medical College, Wah Cantt, PAK

**Keywords:** myelin oligodendrocyte glycoprotein (mog) antibodies, plasmapheresis treatment, high-dose steroid resistance, loss of bowel/bladder control, lower limb weakness, severe progression, atypical symptoms, pediatric-onset multiple sclerosis

## Abstract

This case report presents an atypical instance of pediatric-onset multiple sclerosis (MS) in a 12-year-old male, a demographic less commonly affected by this condition. The patient’s clinical course was marked by severe and progressive symptoms, including lower limb weakness and loss of bowel/bladder control, diverging from the typical relapsing-remitting pattern observed in pediatric MS. Despite initial resistance to high-dose steroid treatment, his condition was ultimately stabilized through plasmapheresis, following the detection of myelin oligodendrocyte glycoprotein antibodies. Unique aspects of this case included the patient’s young age, male gender, and the occurrence of osteopenia, as identified by a dual-energy X-ray absorptiometry scan. This report highlights the variability in MS presentations among pediatric patients and underscores the importance of a personalized, multidisciplinary approach to diagnosis and treatment. It contributes to the growing body of knowledge on pediatric MS, emphasizing the need for heightened clinical vigilance and tailored management strategies in young patients with this complex and lifelong disease.

## Introduction

Multiple sclerosis (MS) is an immune-mediated inflammatory disease that causes episodes of demyelination and neurodegeneration within the central nervous system (CNS). While MS predominantly affects adults, about 3-5% of patients experience symptoms before the age of 18, a condition known as pediatric-onset MS [1]. The estimated prevalence of pediatric MS is relatively low, at 0.2-0.7 per 100,000 children [2]. The typical onset age ranges from 13 to 16 years, and the condition is more frequently observed in girls than boys [3].

Pediatric MS can lead to significant morbidity, including motor disabilities resulting from pyramidal weakness, cognitive decline that affects learning, and various psychosocial challenges. Recent studies indicate that pediatric patients may accumulate physical and cognitive disabilities at a slower rate compared to adults with MS. This slower progression, however, does not diminish the severity and impact of the disease in young patients [4].

The primary objective of this case report is to document a rare case of pediatric-onset MS in a 12-year-old male, emphasizing its diagnostic and management challenges. By presenting the clinical course, treatment outcomes, and insights gained, this report aims to enhance the understanding and management of pediatric MS among clinicians and researchers and to underscore the importance of tailored approaches in the care of young patients with this condition.

## Case presentation

This case report details the diagnosis and management of a rare instance of MS in a pediatric patient. A 12-year-old boy with no significant past medical history and no family history of similar illness presented with a two-week history of progressive lower limb weakness, leading to an inability to walk, and loss of bowel control, resulting in frequent urination and defecation. Additionally, he had a history of delayed developmental milestones, declining academic performance over the past three years, and recent disturbances in sleep and appetite.

On examination, the patient appeared mildly pallor and lethargic, with a Glasgow Coma Scale score of 15/15 and lower limb power of 4/5. Plantar responses were bilaterally downgoing, and the systemic examination was otherwise unremarkable.

**Figure 1:**
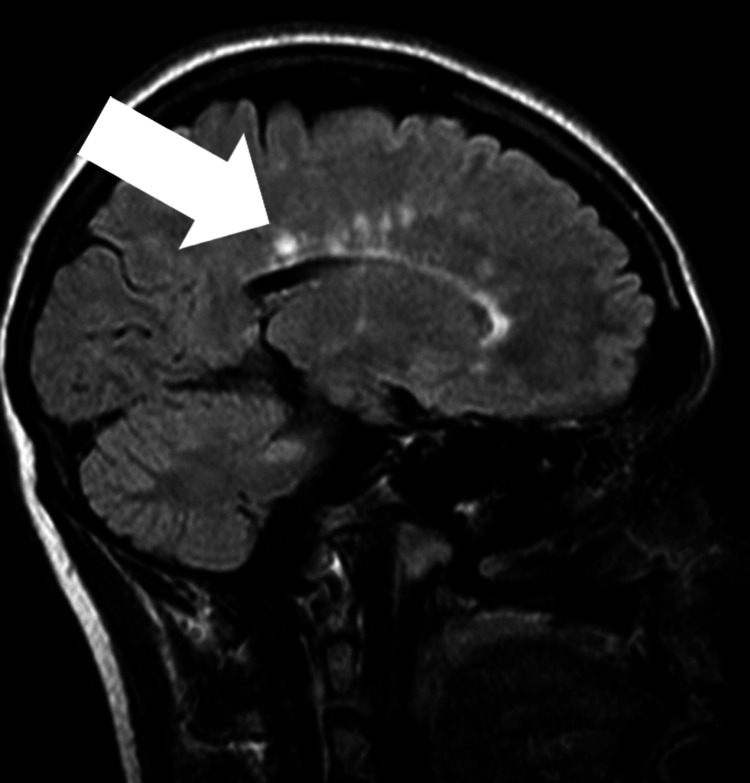
Imaging reveals scattered T2/fluid-attenuated inversion recovery hyperintensities within the brainstem and cerebral white matter, with sagittal plane lesions extending through the corpus callosum, demonstrating “Dawson’s fingers” appearance. The lesions are represented by the arrow.

Diagnostic evaluations revealed elevated erythrocyte sedimentation rate levels, indicating inflammation, and magnetic resonance imaging (MRI) findings consistent with MS, showing features of scattered hyperintensities in the brainstem and cerebral white matter, as evident on T2/fluid-attenuated inversion recovery imaging. Furthermore, sagittal views revealed lesions propagating through the corpus callosum, exhibiting the pathognomonic “Dawson’s fingers” appearance (Figure 1). Further tests showed positive anti-myelin oligodendrocyte glycoprotein (MOG) antibodies and decreased bone mineral density on a dual-energy X-ray absorptiometry (DXA) scan. A summary of initial blood tests is provided in Table 1.

**Table 1:** Initial blood workup of the patient. INR: international normalized ratio; APTT: activated partial thromboplastin time; WBC count: white blood cell count; RBC: red blood cell; HCT: hematocrit; MCV: mean corpuscular volume; MCH: mean corpuscular hemoglobin; MCHC: mean corpuscular hemoglobin concentration; ALT: alanine transaminase; AST: aspartate aminotransferase; ALP: alkaline phosphatase; ESR: erythrocyte sedimentation rate

Coagulation profile	Values	Reference range
Prothrombin time-control	12	10–14 seconds
Prothrombin time-patient	13	Up to 13 seconds
INR	1.08	0.9–1.3
Control time	32	25–35 seconds
APTT	32	Up to 31 seconds
Hemogram		
WBC count	12.5	4–11 × 10^9^/L
Total RBC	5.16	3.8–5.2 × 10^12^/L
Hemoglobin	9.29	13–18 g/dL
HCT	32.3	35–46%
MCV	62.6	77–95 fL
MCH	18.0	26–32 pg
MCHC	28.7	32–36 g/dL
Platelets	507	150–400 × 10^9^/L
Neutrophils	58.3	40–80%
Lymphocytes	31.7	20–40%
Monocytes	6.19	2–10%
Eosinophils	2.58	1–6%
Renal function tests		
Urea	27	10–50 mg/dL
Serum creatinine	0.4	0.5–0.9 mg/dL
Liver function tests		
Bilirubin total	0.5	0.3–1.2 mg/dL
ALT	18	Up to 40 U/L
AST	31	Up to 40 U/L
ALP	171	40-120 U/L
Serum electrolytes		
Sodium	142	135–145 mmol/L
Potassium	4.2	3.5–5 mmol/L
Chloride	106	98–107 mmol/L
Inflammatory markers		
ESR	50	0–25 mm/1^st^ hour

A crucial component in corroborating the diagnosis of MS in this pediatric patient was the analysis of cerebrospinal fluid (CSF). The CSF profile indicated MS, marked by oligoclonal bands exclusive to the CSF and not present in serum, illustrating an immune response confined to the CNS. The immunoglobulin G index was elevated, demonstrating increased intrathecal immunoglobulin production. Although there was a mild increase in lymphocyte count, it remained within the limits typically seen in MS. The protein levels in the CSF were somewhat elevated, yet glucose levels stayed normal. Together with the clinical presentation and MRI results, these findings solidified the diagnosis of pediatric-onset MS, highlighting the necessity of comprehensive diagnostic evaluation.

The patient’s initial management included high-dose intravenous corticosteroids, loperamide, and anticholinergics for bowel control; fampridine to aid in walking, and vitamin D and calcium supplements for bone health. However, upon the recurrence of symptoms and the presence of anti-MOG antibodies, plasmapheresis was initiated, showing improvement after two sessions.

Although the patient experienced symptomatic relief post-treatment, he continued to have difficulty walking. Ongoing physiotherapy was recommended, and he is currently undergoing a total of five plasmapheresis sessions, with a reassessment planned following the completion of treatment.

## Discussion

MS is a chronic, immune-mediated disorder of the CNS that disrupts the flow of information within the brain and between the brain and body. Characterized by periods of demyelination and neurodegeneration, MS presents a significant challenge to both patients and healthcare providers due to its unpredictable clinical course and varied manifestations [1].

While MS is more commonly diagnosed in adults, a small but significant fraction of cases occur in children, with pediatric-onset MS accounting for approximately 3-5% of all MS cases. The incidence of pediatric MS is about 0.2-0.7 per 100,000 children, displaying a distinct demographic pattern that tends to favor females over males and typically presents between the ages of 13 and 16 years [3]. The case of our 12-year-old male patient stands out not only because of his gender but also due to the early age of onset, underscoring the disease’s rarity and the unique challenges it poses in pediatric populations.

Diagnosing MS in children can be particularly challenging due to the overlap of symptoms with other pediatric disorders and the less typical presentation compared to adults. The utilization of high-dose intravenous corticosteroids remains a cornerstone for managing acute exacerbations based on their efficacy in reducing inflammation and promoting rapid recovery of neurological functions [5]. This approach, however, is just one aspect of a comprehensive management strategy that must consider the long-term well-being of the child.

Beyond acute management, the role of disease-modifying therapies (DMTs) cannot be overstated. While not specifically discussed in the initial case presentation, DMTs play a critical role in altering the disease course, reducing the frequency of relapses, and potentially slowing down the progression of disability in pediatric MS. The selection of a DMT must be tailored to each patient’s disease activity, side effect profile, and, critically, their stage of development [6].

For patients with relapsing MS who do not respond adequately to corticosteroids, plasmapheresis offers an alternative therapeutic strategy. By filtering the blood to remove harmful antibodies thought to contribute to the disease process, plasmapheresis can provide a significant improvement in symptoms for those with aggressive MS courses [7]. Our patient’s positive response to plasmapheresis highlights the importance of personalized medicine in MS treatment, emphasizing the need for flexible, adaptive treatment strategies that can be modified based on an individual’s response to therapy.

Managing pediatric MS goes beyond addressing physical symptoms; it encompasses supporting the patient’s cognitive development, psychological well-being, and social integration. Early intervention with rehabilitation services, educational support, and psychological counseling is essential in helping children with MS achieve their full potential. The multidisciplinary approach to care, involving neurologists, pediatricians, rehabilitation specialists, and mental health professionals, is crucial for addressing the complex needs of these patients.

Furthermore, the evolving landscape of MS research continues to shed light on novel therapeutic targets, the role of genetic and environmental factors in disease susceptibility, and the long-term outcomes of children diagnosed with MS. As our understanding of pediatric MS deepens, so too will our capacity to provide more effective, personalized care.

The management of pediatric-onset MS requires a sophisticated understanding of its clinical presentation, a deliberate approach to treatment selection, and a comprehensive strategy for supporting the patient’s overall development and quality of life. Our patient’s journey through the intricacies of MS highlights the vital need for ongoing research, education, and a multidisciplinary approach to care. By sharing these experiences and expanding our collective knowledge, we can enhance outcomes for all children facing the challenges of MS.

## Conclusions

This case report underscores the complexities and atypical nature of pediatric-onset MS through the lens of a 12-year-old boy’s challenging journey. Exhibiting severe and progressive symptoms beyond the common relapsing-remitting pattern, his condition was marked by initial subacute neurological deficits, including lower limb weakness and loss of bowel/bladder control, and a notable resistance to high-dose steroid treatment. The diagnostic puzzle was further compounded by his young age, male gender, and the rarity of osteopenia, as revealed in the DXA scan, in juvenile MS cases. The detection of MOG antibodies served as a critical biomarker, indicating heightened disease activity and guiding the shift toward plasmapheresis. Despite the uncertainties surrounding long-term prognosis, this intervention brought about clinical stabilization and partial functional recovery after the corticosteroid therapy proved ineffective. This case not only illuminates the variable presentations and aggressive potential of pediatric MS but also highlights the indispensable role of a personalized, multidisciplinary approach in treatment. It emphasizes the importance of ongoing research and clinical vigilance to ensure prompt diagnosis and optimal management for young patients navigating the intricacies of this lifelong condition.
